# Dynamic Colon Model (DCM): A Cine-MRI Informed Biorelevant In Vitro Model of the Human Proximal Large Intestine Characterized by Positron Imaging Techniques

**DOI:** 10.3390/pharmaceutics12070659

**Published:** 2020-07-13

**Authors:** Konstantinos Stamatopoulos, Sharad Karandikar, Mark Goldstein, Connor O’Farrell, Luca Marciani, Sarah Sulaiman, Caroline L. Hoad, Mark J. H. Simmons, Hannah K. Batchelor

**Affiliations:** 1 School of Chemical Engineering, University of Birmingham, Edgbaston, Birmingham B15 2TT, UK; CXO348@student.bham.ac.uk (C.O.); M.J.Simmons@bham.ac.uk (M.J.H.S.); 2 Department of Surgery, University Hospitals Birmingham NHS Foundation Trust, Birmingham Heartlands Hospital, Bordesley Green East, Birmingham B9 5SS, UK; sharad.karandikar@heartofengland.nhs.uk; 3 Department of Radiology, University Hospitals Birmingham NHS Foundation Trust, Birmingham Heartlands Hospital, Bordesley Green East, Birmingham B9 5SS, UK; mark.goldstein@heartofengland.nhs.uk; 4 Nottingham Digestive Diseases Centre and National Institute for Health Research (NIHR) Nottingham Biomedical Research Centre, Nottingham University Hospitals NHS Trust and University of Nottingham, Nottingham NG7 2UH, UK; luca.marciani@nottingham.ac.uk (L.M.); sarah.sulaiman@nottingham.ac.uk (S.S.); 5 Sir Peter Mansfield Imaging Centre, University of Nottingham, Nottingham NG7 2QX, UK; caroline.l.hoad@nottingham.ac.uk; 6 Institute of Clinical Sciences, College of Medical and Dental Sciences, Medical School Building, University of Birmingham, Edgbaston, Birmingham B15 2TT, UK; H.K.Batchelor@bham.ac.uk; 7 Strathclyde Institute of Pharmacy and Biomedical Sciences, University of Strathclyde, 161 Cathedral Street, Glasgow G4 0RE, UK

**Keywords:** dynamic colon model (DCM), colon-specific drug formulations, magnetic resonance imaging (MRI), positron emission tomography (PET), in vitro models, dissolution, colon motility, cine-MRI

## Abstract

This work used in vivo MRI images of human colon wall motion to inform a biorelevant Dynamic Colon Model (DCM) to understand the interplay of wall motion, volume, viscosity, fluid, and particle motion within the colon lumen. Hydrodynamics and particle motion within the DCM were characterized using Positron Emission Tomography (PET) and Positron Emission Particle Tracking (PEPT), respectively. In vitro PET images showed that fluid of higher viscosity follows the wall motion with poor mixing, whereas good mixing was observed for a low viscosity fluid. PEPT data showed particle displacements comparable to the in vivo data. Increasing fluid viscosity favors the net forward propulsion of the tracked particles. The use of a floating particle demonstrated shorter residence times and greater velocities on the liquid surface, suggesting a surface wave that was moving faster than the bulk liquid. The DCM can provide an understanding of flow motion and behavior of particles with different buoyancy, which in turn may improve the design of drug formulations, whereby fragments of the dosage form and/or drug particles are suspended in the proximal colon.

## 1. Introduction

Local drug delivery to the colon offers opportunities for effective therapy for a range of conditions, including Crohn’s disease (CD) and ulcerative colitis (UC). European data showed that 2.5–3 million people are affected by Inflammatory Bowel Diseases (IBD), costing healthcare systems 4.6–5.6 billion Euro/year [1]. Approximately 620,000 people are affected in the United Kingdom [2]. Moreover, CD and UC primarily occur in the terminal ileum and the colon. All these findings, alongside the increasing numbers of people affected by IBD [3], have led to a considerable effort to effectively deliver active pharmaceutical ingredients (API) to the colon for local treatment [4].

The oral route is the primary method of administration for the vast majority of the medicines that target the human colon. Most of the modified release (MR) dosage forms have been formulated to release API within the human proximal colon as it provides higher water availability, favoring drug dissolution [5]. Disintegration, dissolution, and absorption/local action are the fundamental processes that take place during the passage of an MR formulation through the human GI tract. Pharmacopoeial disintegration and dissolution tests are used to evaluate and mimic in vitro, the complex in vivo processes. Acceleration of the design, development, and evaluation of MR formulations is vital, and in vitro in vivo extrapolation (IVIVE), when available, can help reduce the number of costly in vivo studies required. However, this requires that in vitro tools can reproduce both the physicochemical characteristics of the gastrointestinal fluids and the hydrodynamics to predict reliably the in vivo performance [6,7,8]. However, the existed pharmacopoeial dissolution apparatuses were designed for batch-to-batch quality control of dosage forms [9,10] without consideration. Hence, oversimplification of the complex and dynamic environment of the human colon [11]. Since hydrodynamics, controlled by the motion of the colon wall, will dictate the shear forces of fluid within the colon and subsequent disintegration, erosion, and dissolution of MR formulations, the development of a biorelevant in vitro colon model for dissolution must accurately replicate colon hydrodynamics.

The DCM was introduced as an anatomically accurate model of the human adult colon, where hydrodynamics can be controlled using a hydraulic system [12]. In our previous published work, the dissolution profile of a high soluble drug (theophylline), released from a hydrophilic MR matrix, was assessed within the DCM using different viscosity fluids as the media [12].

Based on the release technology used to obtain MR, the size, shape, and density of the rate-controlling matrix within the colon lumen may change (e.g., multiparticulates, osmotic pressure pump delivery systems). Therefore, a better understanding of particle motion within the colon under controlled conditions would enable a better understanding of the disintegration, erosion, dissolution, and the residence times of these particles in vivo. This is also of high importance from a Physiologically Based Pharmacokinetic (PBPK) modeling point of view since residence times are normally converted to regional transit times to determine absorption rates and distribution of the dissolved drug between the different absorptive compartments [13].

Visualization of flow and MR product performance is only possible in vivo using MRI or scintigraphy, which is costly, time-consuming, and not practical for product development and optimization. A model that replicates the in vivo colonic environment offers obvious benefits for pharmaceutical development. Information on the in vivo colonic environment is obtained from several sources. Manometry is used to measure the pressure patterns within the colon associated with motility and also to diagnose dysfunctions of the human colon [14,15,16,17,18]. However, translation of the pressure events within the colon to wall motion and the consequent fluid flow is not straightforward, as the viscosity of the lumen will affect the recording pressure amplitude making interpretation of data complicated [12,19]. Given the limitations of conventional manometry, capsule telemetric technologies have been developed, offering a non-invasive assessment of colon motility [20,21]. However, the magnetic capsule used is a high density (sinking), large-solid marker and these attributes make interpretation of the resulting data only applicable to similar products (large, high-density particles), and thus, difficult to correlate the measured pressure events and fluid displacement to the “usual” colon [20].

MRI imaging of the colon provides insights into the undisturbed colon environment, including wall motion and fluid distribution [5,22,23]. MRI is non-invasive; thus, there is no disruption to the physiological, physical state of the colon. By visualization of colonic wall motion and, in particular, segmental (haustral) motion, information about the rate and extent of occlusion in each haustrum can be obtained. Extraction of the motility patterns from in vivo MRI images provides an opportunity to reproduce colonic wall motion in a biorelevant in vitro model. This biorelevant physical model will allow exploration of the relationships between wall motion, intraluminal pressure amplitudes, viscosity, volume, particle motion, and hydrodynamics within the colon that is not possible in vivo.

Furthermore, knowing/exploring this interplay between those factors, patients with specific profiles (i.e., motility patterns, fluid volumes, and so on) could be treated with the appropriate drug formulation, which will perform better under those patient-centric conditions. It has been shown that gastrointestinal transit times (GTT) are increased in patients with UC [24], whereas high variability in GTT has been observed in patients with severe UC [25]. In particular, the colonic transit times in healthy volunteers have a range of 7–20 h, whereas there is a much wider range (2–97.7 h) in patients with active UC [25]. Thus, formulations with different release characteristics may benefit from being tailored to individual patient phenotypes within the same disease population according to the severity of the disease.

In this work, cine-MRI of the cecum-ascending colon in healthy human adult subjects was used to derive motility patterns of the colonic wall and inform the development of relevant motility patterns within a Dynamic Colon Model (DCM). Positron Emission Tomography (PET) and Positron Emission Particle Tracking (PEPT) were used to visualize the fluid flow and the particle motion within the DCM, respectively. An understanding of fluid flows in an anatomically accurate model of the colon will provide information that will aid the design of future MR formulations.

## 2. Materials and Methods

### 2.1. Materials

Sodium CarboxyMethylCellulose (NaCMC_MW:700,000_) was purchased from Sigma, St. Louis, MO, USA. Silicone rubber (Polycraft T-15 RTV) was purchased from MB Fibreglass, Newtownabbey, UK. The School of Physics and Astronomy at the University of Birmingham, Birmingham, UK, provided an 18F radionuclide solution and 18F labeled ion-exchange tracer to run the PET and PEPT experiments, respectively.

### 2.2. The Dynamic Colon Model (DCM)

The DCM, a biomechanical engineering model of the adult human proximal colon, was designed and developed in the School of Chemical Engineering at the University of Birmingham, Birmingham, UK (Figure S1). The clinicians in the study team provided relevant anonymized abdominal static MRI images to inform its development. Full details of the development and the main features of the model can be found in the supplementary material. Briefly, the DCM is comprised of 10 haustral units (Figure S2A) with a total length of 200 mm (209 ± 47 mm length of the cecum-ascending region in humans [26]). The total volume of this tubular model is 290 cm^3^, which is within the physiological values with range 76–390 cm^3^ [27]. Each unit was connected to a syringe, which was controlled by a stepper motor. Backward and forward motions of the syringe cause inflation and deflation of the membrane. Synchronization of the units was performed using a computer-controlled hydraulic system (Figure S2B). 

### 2.3. In Vivo cine-MRI of Cecum-Ascending Colon in Healthy Human Subjects

**Figure 1:**
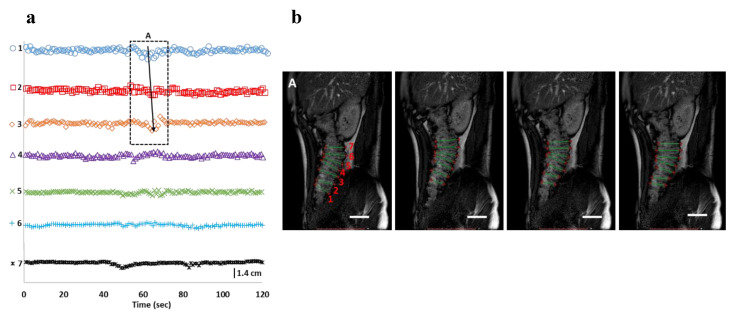
Baseline haustral activity in the cecum-ascending region, detected by the cine-MRI technique. (**a**) Tracking of changes of the length of each haustrum (i.e., length changes of the straight lines in MRI images, cm) as a function of time; (**b**) Corresponding MRI frames of the wave (A) detected; The corresponding cine-MRI can be found in Video S1; The arrow shows the direction of the propagating contractions; the numbers in (**a**) reflect the haustrum numbering in MRI frames (**b**); open bar (6 cm)**.**

**Figure 2:**
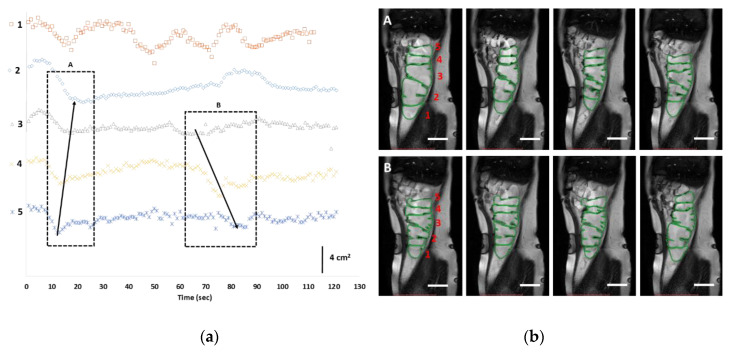
Haustral activity in the cecum-ascending region, detected by the cine-MRI technique after oral PEG electrolyte bowel preparation. (**a**) Tracking of changes in the surface area (cm^2^) of each haustrum as a function of time. (**b**) Corresponding MRI frames of the two waves (A and B) detected. The corresponding cine-MRI can be found in Video S2. The arrows indicate the direction of the propagating contractions; the numbers in (**a**) reflect the haustrum numbering in MRI frames (**b**); open bar (6 cm).

The University of Nottingham provided anonymized MRI anatomical images and cine-MRI data sets of the human cecum-ascending colon. These images originated from pre-existing studies published elsewhere [23,26]. Ethical approval was in place for further research use of these data. MRI imaging of the cecum-ascending region was performed in healthy subjects using different stimulants [23,26]. Briefly, healthy volunteers received different stimulants orally, namely maltose or PEG electrolyte solution. The colonic motility scan comprised a sagittal, balanced turbo field echo (bTFE) image slice positioned by the radiographer centrally through the ascending colon (AC). The field of view was displayed as a 384 × 384 matrix with 0.86 mm × 0.86 mm in-plane resolution. Other parameters set were a slice thickness of 15 mm, a flip angle of 50°, a repetition time/echo time of 2.4/1.2 ms, and an acceleration factor of 2.5. The cine dataset was acquired at a frame rate of 1 image per second, for 2 min. MRI images were analyzed with ImageJ (v. 1.52a). With regards to baseline conditions, tracking of the colon motility was performed (Figure 1a) by measuring changes of the length (cm) of locations on opposing points of the wall of each haustrum joined by straight lines (Figure 2b). Under stimulated conditions, the distention of the wall and the contrast ratio, due to the presence of higher fluid volumes in the ascending colon, allowed the region of interest (ROI) of each haustrum to be easily identified. Therefore, changes in the surface area (cm^2^) of each haustrum was monitored as a function of time. Any reduction of the surface area or the length of the straight lines implies contraction, whereas an increase implies relaxation. A motility index was determined as described elsewhere [26]. The colon was divided into five segments, and the motility index score was calculated every 20 s using:

Motility index (segment × s) = (Dr.No_seg_)*_1_* + … (Dr.No_seg_)*_i_* (1)

where Dr is the duration of the contractions within 20 s, No_seg_ is the number of the segments of the ascending colon showed motility and *i* = 1, 2,…,6 (i.e., 120/20 = 6 measurements). As an example, if for the first 20 s, the whole ascending colon shows motility and for the following 20 s only the one-fifth shows motility, then the motility index is calculated as followed

Motility index (segment × s) = (20 × 5) + (20 × 1) = 120 (segment × s)

### 2.4. Reproducing the Wall Motion Patterns in DCM Observed in Cine-MRI Study

The current design of the DCM allows haustra to contract (i.e., membrane inflation) and relax (i.e., membrane deflation) while the semilunar folds have a fixed position, unlike in reality. However, the synchronized haustral contractions/relaxations in DCM provide a semi-continuous motion of the wall, in the antegrade or retrograde direction, which can overcome, to some extent, this limitation. Haustral activity, observed in cine-MRI, was used as a case study to link the wall motion of this type of motility and link to the intraluminal hydrodynamics within the DCM. ImageJ (v. 1.52a) was used to analyze selected images from cine-MRI to determine a physiological range of occlusion degree (reduction of the length or the surface area of a single haustrum). The degree of occlusion of each unit of the DCM reflects this range accordingly. Technical details about operational background of DCM model can be found in supplementary material. In particular, Figure S3 shows the time courses of membrane oscillations whereas Figure S4 shows the calibration of the DCM.Figure S5 shows the interface of the software used to control and synchronize DCM units.

### 2.5. Fluids Used in PEPT and PET Experiments

Both hydrodynamics and particle motion was examined in fluids of different viscosity. Fluids with varying concentrations of NaCMC_MW:700,000_ (*w*/*w*) were used to mimic the increase of the viscosity due to the re-absorption of water, which takes place in the human colon. In particular, 0.25, 0.50 and 0.75% NaCMC (*w/w*) were used, corresponding to low (denoted L), medium (denoted M), and high (denoted H) apparent viscosity. The concentrations of NaCMC were in accordance with previous studies published to assess the impact of the viscosity on the dissolution profile of drugs contained in a hydrophilic dosage form targeting the human colon [18,19,20]. Sodium chloride (3% *w/w*) was added to the fluids, and then the flow rheology of the media was measured using Discovery Hybrid Rheometer (TA Instruments), coupled with a 40 mm diameter, 4° cone, and plate geometry to make particles neutrally buoyant. A Herschel–Bulkley model described the flow rheology of the fluids:

(2) $$\tau =\tau_{\gamma }+K{\dot{\gamma }}^{n}$$

where *τ* is the shear stress, *τ*_γ_ is the yield stress, $\dot{\gamma }$ is the shear rate, *Κ* is the consistency index, and *n* is the power-law exponent (< 1). 

### 2.6. Positron Emission Particle Tracking (PEPT) System

Positron Emission Particle Tracking (PEPT) [27,28], developed at the University of Birmingham, was used to assess fluid patterns within the DCM tube. The technique relies on the Lagrangian tracking of a single radioactive tracer particle. The particle, an ion-exchange resin particle of diameter 0.250–0.300 mm doped with 18F, was provided by the School of Physics at the University of Birmingham. 18F is a positron emitter; the annihilation of the positron produces two incident back to back γ rays. These are detected using a DAC Forte PET scanner, and through the detection of multiple events, the position of the particle is triangulated. The method enables the location of the particle to be detected with sub-mm resolution several times per second, where the precise resolution is a function of the activity of the particle [28]. The half-filled DCM tube was placed between the two detectors within the scanner, and a radioactive tracer particle was introduced in the first segment. The half volume (i.e., 100 mL) was chosen as the closest to the overall volume that a dosage form is likely to be exposed to during its passage through the human colon [29], and it is also similar to the volume typically used in mini-USP 2 [12].

The tracer was enclosed in a cylindrical polystyrene particle (3 × 3 mm) by drilling a hole of 1 mm Ø and reheating it to seal it. A similar procedure was followed for a polypropylene particle of the same dimensions. Thus, the polystyrene particle would be expected to be neutrally buoyant (n.b) due to its higher density compared to polypropylene. The latter will float, expecting to track any motion at the liquid–air interface.

Particle relaxation time is the time of a particle to respond to fluid motion. This parameter was determined for both floating and n.b particles, using:

t_o_ = (*ρ*_d_ d_d_^2^)/18 *μ*_A_ (3)

where *ρ_d_* is the particle density (kg m^−3^), *d_d_* is the particle diameter (m), and *μ*_A_ is the apparent viscosity (kg m^−1^ s^−1^) of the fluid at $\dot{\gamma }$ = 10 s^−1^. Equation (4) was used to determine the value of Reynolds number, Re, of the flow in the DCM tube.

Re = (*d*_h_ ū *ρ*)/*μ*_A_ (4)

where $\bar{u}$ is the flow velocity (m s^−1^), *d*_h_ is the hydraulic diameter (m), and ρ is the fluid density (kg m^−3^).

The data acquisition algorithm used to provide the location of the particle in Cartesian co-coordinates at discrete time intervals was published elsewhere [30]. The data must be reconstructed to identify the position of the radioactive tracer with respect to the DCM tube coordinates. Before each motility pattern, the tracer was positioned at the center of the first segment to obtain the *x*, *y*, and *z* coordinates. In all runs, the particle was placed approximately at the same position, i.e., *x* = 0 ± 2 mm and *y* = 0 ± 3 mm. Unlike the floating particle, the zero position of the n.b particle, with regards to the *z*-axis, was 5 ± 2 mm below the surface of the fluid. The Savitzky–Golay smoothing/filter method [31] with a second order polynomial fitting was used for smoothing the PEPT data and ensuring a low signal-to-noise ratio. Considering that the size of both particles was 3 mm, only movements of at least 10 mm along the tube (i.e., *x*-axis) were assigned as propulsive displacements and used for further analysis. Equation (5) was used to analyze the propulsive velocities in the DCM tube.

*U_x_* = Δ*x*/Δ*t* (5)

where Δ*x* is for displacements ≥ 0.01 m.

Only a complete travel of the radiotracer along the DCM tube was considered as a full pass. After reaching the very end of the tube, the radioactive particle was collected and placed back to the initial position. Then, a new series of motility patterns were applied. In total, 30 passes of the tracer were performed in each viscous media used.

### 2.7. Positron Emission Tomography (PET) to Visualize Fluid Motion within the DCM Tube

Propulsive and non-propulsive events within the DCM tube were tracked using PET under fixed motility patterns, and the relationships between wall motion and contents’ movements were examined. The DCM tube was half-filled (i.e., 100 mL, refer to Section 2.5) and positioned between two positron emission detectors (DAC Forte PET scanner, refer to Figure S6). Then, 1 mL of ^18^F labeled aqueous solution was injected through a hole of 10 mm Ø of the first segment, representing the human terminal ileum. PET images were recorded at two acquisition frequencies, at 10 s frame^−1^ (as reported in the literature [32]) and at a 1 s frame^−1^ and synchronized with the membrane motion.

### 2.8. Statistical Analysis

Kruskal–Wallis one-way ANOVA on ranks statistical analysis was performed to compare multiple groups with non-normal distribution and unequal size PEPT data. Statistically significant differences were considered at *p* < 0.05. The statistics were calculated using Sigmaplot (v. 14, Systat Software Inc, San Jose, CA 95131 USA, 2018).

## 3. Results and Discussion

### 3.1. Determining Haustral Activity in the Cecum-Ascending Colon Using Cine-MRI

**Figure 3:**
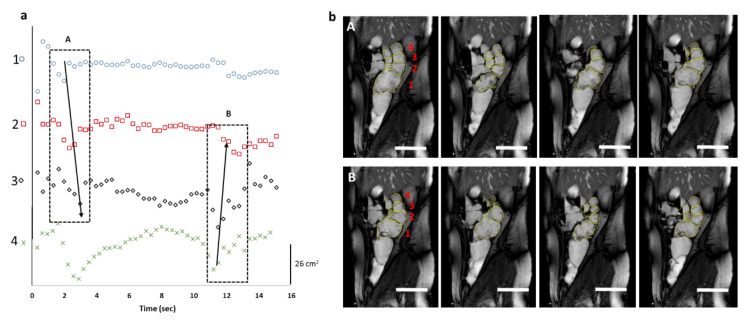
Haustral activity in the cecum-ascending region, detected by the cine-MRI technique after oral maltose bowel preparation. (**a**) Tracking of changes in the surface area (cm^2^) of each haustrum as a function of time. (**b**) Corresponding MRI frames of the two waves (A and B) detected. The corresponding cine-MRI can be found in Video S3. The arrows indicate the direction of the propagating contractions; the numbers in (**a**) reflect the haustrum numbering in MRI frames (**b**); open bar (4 cm).

Analysis of anonymized MRI images revealed differences in motility index, occlusion degree, occlusion rate, the direction of the wave, and the velocity of the propagating contractions (Table 1). Baseline conditions showed the lowest activity of the wall motion (motility index 120 ± 50 segment x s). Most of the wall activity appeared as random isolated haustral contractions of small occlusion degrees (Figure 1a). A short (3.9 cm) antegrade propagating wave was detected (Figure 1a (1st–3rd curve); the entire cine-MRI for the baseline condition can be found in Video S1. Under stimulated conditions, the distention of the colon wall and contrast ratio, due to the presence of high water volumes, was sufficient enough to allow positioning ROIs for each formatted haustrum (Figure 2b and Figure 3b). Therefore, in this case, surface area changes of the ROIs were tracked as a function of time, instead.

Unlike the baseline conditions, retrograde waves, started from hepatic flexure, and these (Figure 2a,b) caused forward and backward fluid motion (Video S2). Interestingly, differences in motility after different stimulants were observed. Elevated motility was observed in cecum-beginning of ascending colon (Figure 2a, 1st curve) prepared with PEG-electrolyte, whereas higher motility was observed at the middle-to-the-end of the ascending colon when maltose was used as a stimulant (Figure 3a, 4th curve).

Independent of the stimulant, the time to reach the maximum occlusion degree was always shorter (8.64 ± 10.4 s) compared to the time needed to return to the initial state (33 ± 14.3 s), giving a ratio of 3.82 (relaxing/contracting); in the baseline condition, the ratio was ~1.1. Significant differences were observed between stimulated and baseline conditions for the travel distance and travel velocity of the antegrade waves (Table 1). Under stimulated conditions, the travel distance was 5.3 ± 1.4 cm and in baseline was 3.9 cm, whereas the travel velocity was 2.2 ± 3.3 cm s^−1^ and 0.98 cm s^−1^, respectively. This translates in 1.3 and 2.2 folds increase in travel distance and travel velocity, respectively, after stimulation of the human colon.

**Figure 4:**
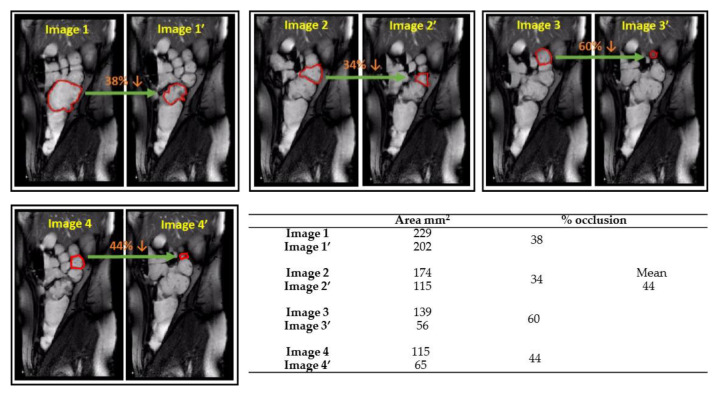
Haustral occlusion degree quantified by the decrease of the area of ROI (Region of Interested); Image analysis conducted with using ImageJ (v. 1.52a); the frames are depicted from Video S3.

Increased motility was observed under stimulated conditions (motility index 320 ± 138 segment x s), which was significantly higher than the baseline. Figure 4 shows an example where MRI images were analyzed to determine the occlusion degree of different haustrum in one individual, showing a wide range (34–60%); the corresponding cin-MRI form which images depicted can be found in Video S3 In general, higher occlusion degrees (i.e., decreases in surface area of the haustra) were observed in stimulated conditions (59 ± 18%), implying stronger contractions, compared to the baseline (18 ± 10%). The number of antegrade waves was also higher (9 ± 1). 

Another parameter determined to assess differences between baseline and stimulated colon motility, was the occlusion velocity (cm s^−1^). This parameter will also help to set up the DCM, as it is explained in the following section. The occlusion velocity was found to be 3.6 ± 0.17 (cm s^−1^) and 0.14 ± 11 (cm s^−1^) under the stimulated and baseline conditions, respectively, giving a 2.6 fold increase.

The quantitative insights provided by MRI into haustral activity are unique. The motor function of this part of the anatomy is particularly challenging to observe without disturbing the physiology with invasive intubation techniques while ionizing radiation damage limits the use of X-ray based medical imaging techniques. The MRI imaging planes displayed large sections of the ascending colon with spatial resolution in the millimetric range, allowing the measurement of propagating contractions and surface area changes under different conditions. The data here were analyzed using freely available image analysis software. It is worth mentioning that more sophisticated semi-automated analysis methods have been recently developed and reported [22]. The use of machine learning is developing in this field and could make this type of data analysis quicker and suitable, for example, for population- and disease-based and investigations and modeling.

### 3.2. Replicating the Haustral Motility in DCM

Table 1 shows the values of each parameter obtained from the analysis of MRI images used to set up the motility pattern in the DCM. The motility profile of DCM lies between the baseline and stimulated conditions examined in vivo. Repeated haustral contractions were applied with a travel velocity of 2 cm s^−1^ with a 10 s time delay between them to ensure the fluid became static before the next wave of contractions occurred. This repetition gives four waves every 2 min and, therefore, a motility index score of 240. Since retrograde waves were observed only under stimulated conditions, antegrade waves were mainly applied in this work.

**Figure 5:**
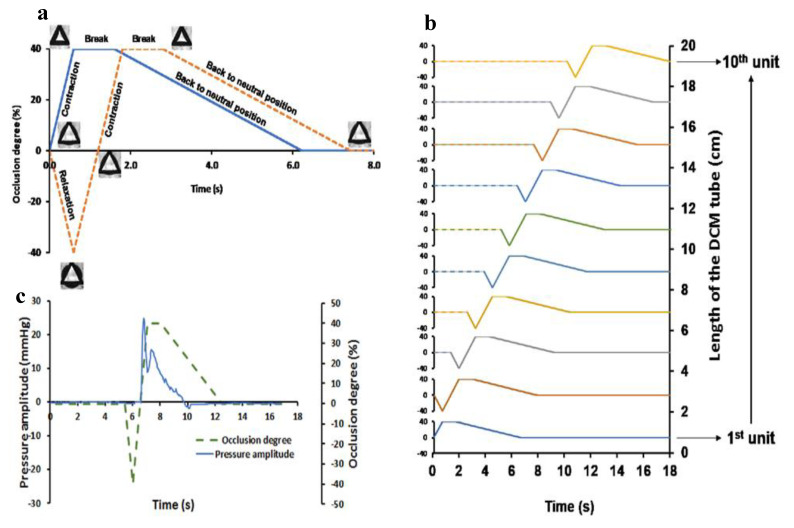
Haustral contractions in DCM. (**a**) Profile of contraction-break-relaxation cycle of each DCM unit; (**b**) wave of propagating haustral contractions; (**c**) membrane motion and the corresponding intraluminal pressure profile within a DCM unit recorded by solid-state manometry.

The profile of the wave of the DCM wall was set up as follows:

(1) The membrane of the first segment was inflated, whereas, in the second, it was deflated at the same speed, 1.6 cm s^−1^.
(2) When the first segment reaches the maximum degree of luminal occlusion, it stays at this position for 1 s before going back to its neutral position at a lower speed of 0.35 cm s^−1^ (Figure 5a).

The slower speed of the wall to return to its position was applied to mimic the viscoelastic properties of the colon wall and to match the ratio obtained from the analysis of the MRI images between the time to reach the maximum occlusion and the time it took to go back to the initial state. The travel distance of the wave in the DCM was 20 cm, i.e., the entire length of the model (Figure 5b). Although such long travel distances were not observed in the in vivo data sets analyzed, for practical reasons, the travel distance was set as the length of the DCM tube as this allowed a better understanding of the relationship between wall motility and fluid/particle motion, as more data could be collected within the experimental time. It is worth noting that in vivo, the colon wall relaxes actively based on the mechanical stimulus generated by the pressure applied on the wall by the bolus [33]. To what extent the wall relaxes depends on the pressure applied on the wall which is related to many parameters including the fluid volumes, fluid viscosity, pressure distribution between the contracting and the relaxing region, and how the Enteric Nervous System (ENS) reacts to this mechanical stimulus [33,34]. These complex phenomena cannot be reproduced easily in vitro. Therefore, to semi-mechanistically reproduce the so-called “intestine law” (i.e., coordinate contraction/relaxation of the smooth muscle in vivo) in the DCM when the first unit contracts, the following unit expands at the same speed, i.e., 1.6 cm s^−1^. This approach allows a physiological luminal pressure, measured by a solid-state catheter, to be obtained (Figure 5c), i.e., no negative pressure due to actively sucking the membrane of the DCM units by a negative pressure applied via the syringes. Moreover, as mentioned above, the lower speed at which the membrane is reverted to its initial position causes a tailing on the pressure profile. This is similar to in vivo observations with high-resolution manometry [14].

### 3.3. Positron Emission Tomography (PET)

Relationships between membrane motion and fluid flow were investigated by performing PET in different viscous media. Unlike the dynamically changing in vivo colonic environment, the conditions were fixed in this in vitro study to understand the interplay between the wall motion, fluid flow, and viscosity.

**Figure 6:**
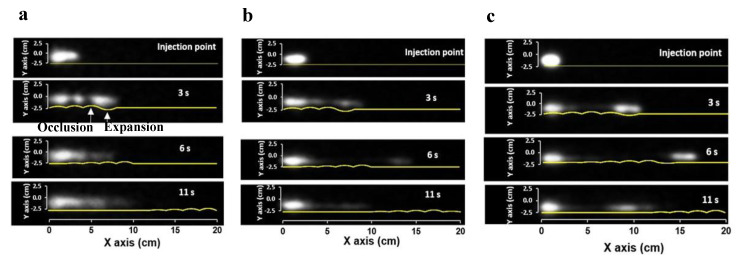
Time series of Positron Emission Tomography (PET) images, captured rate 1 fps, and the corresponding membrane status at the current time interval. (**a**) 0.25% NaCMC (*w/w*); (**b**) 0.50% NaCMC (*w/w*); (**c**) 0.75% NaCMC (*w/w*). The degree of luminal occlusion was 40%; wave speed 0.02 m s^−1^; DCM tube filled 50% with fluid.

Figure 6 shows the time series of PET images of a single wave of haustral contractions in 0.25% (Figure 6a), 0.50% (Figure 6b), and 0.75% (Figure 6c) NaCMC (*w/w*), respectively. The membrane status of the DCM at each time point is also presented. It should be noted that the image capture rate in PET was much higher (i.e., one fps) compared to in vivo studies (one frame per 10 s; [32]). This allowed a more detailed temporal analysis of the flow behavior as a function of the wall motion. The results showed that, at low viscosity (Figure 6a), the movement of the fluid followed the motion of the wall for the first 3 s after which the fluid flowed back even though the wall wave motion was still traveling forward towards the end of the tube. This occurred because the level of fluid was raised close to the wall of the rigid siphon without passing over it. This resulted in a strong backflow due to gravity and relaxation of the wall back to its neutral position. This phenomenon was also observed in cine-MRI under stimulated conditions (Video S2). This mechanism led to poor overall forward propulsion of the contents. This backflow effect was less significant in the more viscous media; Figure 6b shows that a portion of the radioactive tracer covered a longer distance (see the frame at 6 s) before it moved back. In contrast, for the most viscous fluid (Figure 6c), the radioactive tracer traveled the entire length of the tube before it moved back, ending in the middle of the tube. In addition, PET images showed that the portion of the tracer, traveling with the wave-front of the membrane, was located inside the expanding haustrum. Furthermore, especially for low viscosity media, the tracer appeared in the form of pockets located within the haustra during the wall motion. However, these pockets disappeared after the completion of the wave as the fluid was redistributed due to backflow. This phenomenon occurred less frequently in more viscous fluids.

**Figure 7:**
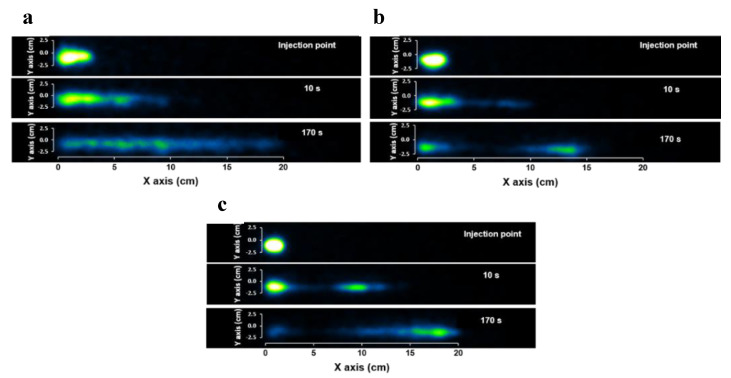
Time series of PET images captured at 10 s frame^−1^. (**a**) 0.25% NaCMC (*w/w*); (**b**) 0.50% NaCMC (*w/w*); (**c**) 0.75% NaCMC (*w/w*). The degree of luminal occlusion was 40%; wave speed 0.02 m s^−1^; DCM tube filled 40% with fluid. The first frame corresponds at zero time, the second on after 10 s where a single wave has been applied and the last frame captured after 170 s, i.e., at the 9th wave. Between each wave, there was a 10 s delay to ensure stagnant fluid before applying the next one.

Figure 7 shows the PET images with a longer recording time interval (i.e., 10 s frame^−1^). This recording time was the same used in previous in vivo studies [32]. Since the wave lasted for 10 s, the PET images were captured after the end of the wave motion. Thus, the distribution of the tracer is due to the motility event, which occurred before the image was captured. The PET images showed that for the low viscosity media (0.25% NaCMC *w/w*), the tracer was distributed throughout the DCM tube after nine repeated waves (last frame in Figure 7a). However, for the higher viscosity fluids, the distribution of the tracer was much less uniform (Figure 7c). Two spots with high tracer intensities were formed, showing that plug flow and less mixing occurred in high viscosity media. The final distribution of the tracer was affected by the ‘to and fro’ motion of the fluid. These movements are a combination of the forward propulsion of the fluid and the backflow after the wave has passed. The extension of the ‘to and fro’ depends on the viscosity of the fluid.

In all PET experiments, a significant amount of tracer remained at the beginning of the tube (i.e., within the caecum and close to terminal ileum), something which has also been observed in the in vivo scintigraphy studies [32,35]. This is probably because the propulsion of the fluids might not be strong at the beginning of the human proximal colon contraction, as the propagating wave is not yet fully developed.

It should be noted that these observations might differ significantly if PET images captured using different fluid volumes, though, with the same motility pattern. Nevertheless, the behavior of the colon wall depends on the mechanical and chemical stimulus and the disease state [33]. Thus, propulsion and mixing of the contents should carefully be assessed when changing only the volume but keeping the same motility pattern.

### 3.4. Assessing Particle Motion in DCM Using PEPT

PET images showed that the dissolved radiotracer is not distributed uniformly along the DCM tube and that the distribution is an interplay of wall motion, viscosity, and “to and fro” fluid flow. It can be assumed that dissolved drug particles can be affected similarly. During the disintegration of a tablet or drug released from an osmotic pump formulation, particles of different size and buoyancy are suspended in the luminal environment. In this work, a floating and a neutrally buoyant particle were used to investigate how the viscosity and the hydrodynamics upon wall motility affect their motion. This provides insights into the distribution of those particles and potentially the distribution of the dissolved drug within the region of interest.

Table 2 shows the relaxation times of the floating and neutrally buoyant particle used in this study with respect to the relaxation times of the magnetic pills used to monitor in vivo colonic movements [20,21]. The particle tracers used in this study had relaxation times an order of magnitude smaller than the magnetic pills used in in vivo studies (e.g., 0.065 s compared to 0.378 s, Table 2). In particular, the floating particle had relaxation times of 0.059, 0.004, and 0.002 s, whereas values for the n.b particle were 0.065, 0.005, and 0.003 s for L (Low viscosity, 8 mPa·s), M (Medium viscosity, 100 mPa·+s), and H (High viscosity, 200 mPa·s) fluids respectively. The magnetic pill used by Hiroz et al. (2009) had relaxation time values of 0.378, 0.028, 0.015 s for L, M, and H fluids, respectively. The wide range of relaxation times of particles used in this study, coupled with the wide range of viscosity of fluids, provided greater insights into colonic flow compared to the in vivo study with a single magnetic pill. The pill used by Hiroz et al. [20] might be expected to follow the flow somewhat only when the viscosity of the colonic fluids is sufficiently high (due to the density of the magnetic pill). Mark et al. (2019) used a magnetic capsule which was bigger but lighter (dimensions: 21 mm × 8 mm, density 1.6 g cm^−3^) [21] from the capsule used in Hiroz et al. (dimensions: 18 mm × ø 5.5 mm, density 1.8 g cm^−3^). The corresponding relaxation times for the capsule used by Mark et al., were 0.178, 0.013, and 0.007 s for the L, M, and H fluid, respectively. These shorter relaxation times imply that the capsule will better follow the flow at lower viscosities compared to the capsule used by Hiroz et al. [20]. Comparable relaxation times between the tracers used in this study and the capsule in Mark et al. were obtained only at the high viscosity media (0.002 s compared to 0.007, Table 2). It should be pointed out that in vivo studies are typically limited to single formulations examined in simple conditions, whereas the DCM can be used to investigate multiple formulations in several conditions.

**Figure 8:**
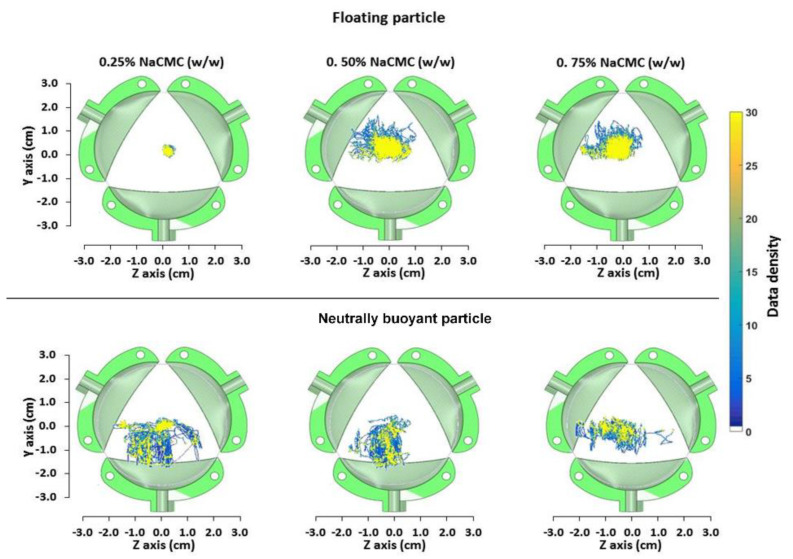
The data density of the axial displacements of floating and neutrally buoyant particles across the cross-section of the Dynamic Colon model tube, in different viscous media. Data density derived after compressing in a single data matrix all the passes of each particle through the DCM units.

Figure 8 shows the data density plots of the axial displacements of the floating and n.b particle in each of the media. In media L, the motion of the floating particle was limited around the center of the DCM tube. This meant that the particle followed the small changes in the level of the wave height during the wall motion without significant oscillations in the *y* and *z* directions. However, considering the wider area covered by the n.b particle, it seemsed that the mixing in the bulk fluid was more intensive. However, increasing the apparent viscosity using fluids M and H led to a significant change in the behavior of the floating particle, with large oscillations observed. Indeed, in fluid L, the movements of the floating particle around the center of the tube were ±5 mm in both axes. Meanwhile, in fluid M and fluid H, the oscillations were approximately ±10 mm in the *y*-direction and ±15 mm in the *z*-direction for both. It is a possibility that for low viscosities, the fluid is more prone to a sloshing motion, whereas for high viscosities, this motion is damped, and the fluid moves in a plug-like manner (plug flow). This could explain why the values of the wave height for the M and H media were closer to the maximum displacement (12 mm) of the membrane during the contraction.

In case of the n.b particle, the apparent viscosity seemed to affect the efficiency of the mixing of the bulk fluid, since a relatively smaller area was occupied by the tracer in the M and H media compared to fluid L. Unlike fluids L and M, the particle movements in fluid H were mainly close to the surface of the fluid and along the *z*-axis. In contrast, in the other two viscous media, the tracer had almost reached the bottom of the haustrum.

However, it must be noted that the behavior of the n.b particle also depended on its location along the *y* and *z*-axes after each propagating wave. Thus, if the n.b particle is located, e.g., 5–10 mm below the surface of the fluid and after a single or several waves it has been relocated closer to the surface, then it will follow a different path compared to the circumstance in which the particle remains at its original depth. Indeed, the data for the n.b particle showed that a significant reallocation of the tracer occurred after the contraction of the first unit (Figure S7). However, in the case of fluids L and M, the n.b particle remained sufficiently submerged in the fluid compared to fluid H.

**Figure 9:**
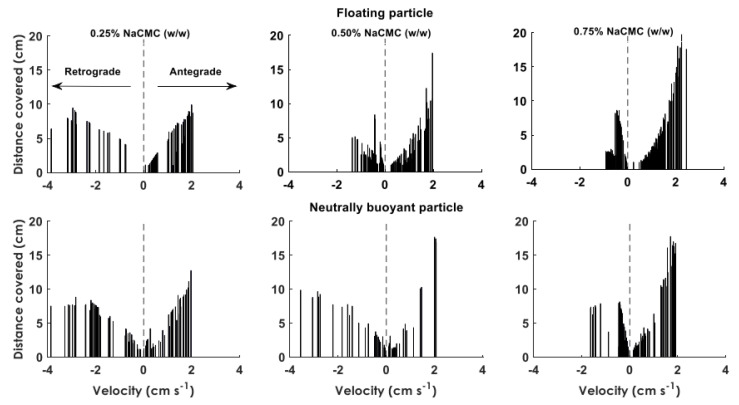
Analysis of distances covered at different velocities based on tracking data of the floating and neutrally buoyant particles obtained in different viscous media.

Figure 9 shows the distance covered by the tracer particles at different velocities, in both retrograde (negative velocity) and antegrade (positive velocity) directions. In fluid L for both floating and n.b particles, a strong backflow is observed with a similar magnitude to the forward motion. The distances covered at different velocities were within the same range 1–9.5 cm. This was also revealed from the high negative velocities observed for both particles, reaching values ≈4 cm s^−1^. With regards to fluid M, the range of the distances for the floating particle covered in retrograde direction was within the range of 1–8 cm for velocities between 0.2–1.5 cm s^−1^. Meanwhile, the distances covered in antegrade direction were in the range of 1–18 cm for velocities between 0.12 cm s^−1^. However, the n.b particle experienced a backflow similar to that observed for fluid L. In the case of fluid H; the results showed the least backflow of the fluid with retrograde velocities of 0.2–1 cm s^−1^ for the floating particle and 0.2–1.7 cm s^−1^ for the n.b particle. The range of the antegrade displacements was between 1–19 cm and 1–17 cm for the floating and n.b particle, respectively. In addition, most of the retrograde displacements were <0.05 cm for the floating and n.b particle, respectively. Furthermore, the maximum velocity for the antegrade displacements was analogous to the wave speed (2 cm s^−1^), with the only exception being the data obtained in fluid H for the floating particle where the velocities were slightly higher than the wave speed (2.3 cm s^−1^). All the individual passes of the particles in different viscous media at y vs x axis can be found in Figure S7 and displacements at x axis vs time can be found in Figures S8–S10.

**Figure 10:**
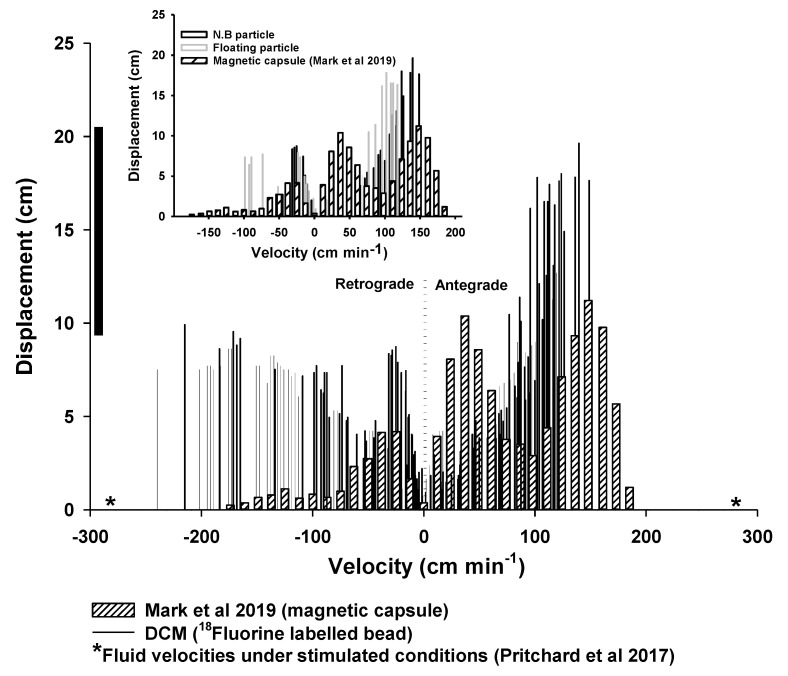
Comparison of propulsive velocities observed in the DCM (^18^Fluorine labeled bead) for both floating and neutral buoyant particles in all different viscous media and magnetic capsules [21]. The inset graph presents the histogram of distances covered at different velocities for the floating and neutral buoyant particle obtained at 0.75% NaCMC (*w/w*) and those of the magnetic pill [21] The solid line next to the *y*-axis of the main plot shows the range of the displacements of the magnetic capsule in Mark et al. (2019). The asterisk (*) in the x-axis indicates the fluid velocities (≥288 cm min^−1^) observed in ascending colon under stimulated conditions using MRI tagging [36].

Previous in vivo analysis of the colonic motility using a magnetic pill revealed a large spectrum of distances covered at different velocities [20,21]. A direct comparison with the in vivo data was not straightforward since the particles in this study had relaxation times that were an order of magnitude smaller than those of magnetic capsules (Table 2). In addition, the wall motion, as well as the viscosity, the volume, and the density of the fluid, were predetermined and fixed in the experiments presented in this work in contrast to the dynamically changing in vivo colonic environment. Hinoz et al. [20] and Mark et al. [21] have reported similar quantitative data to this study. As mentioned above, the relaxation times of the magnetic capsule used in Mark et al. were shorter compared to Hinoz et al., whereas, at the most viscous media (i.e., 0.75% NaCMC), they were comparable to the beads used in this study (Table 2). Knowing the limitations of a direct comparison, Figure 10 shows the histogram of distances covered at different velocities in the DCM and Mark et al. study. It has to point out that the velocities in Figure 10 from the Mark et al. study were from all the regions of the colon. The solid line next to the *y*-axis of the main plot showed the range of the displacements (8.7–20.8 cm) of the magnetic capsule observed within the cecum-ascending region, whereas the corresponding range in the DCM was 1–19 cm. The velocities experienced by the particles during the wall motion of the DCM tube never exceeded 2.2 cm s^−1^ for antegrade displacements, which were relatively close to values reported in the in vivo studies, i.e., 1.7 cm s^−1^ [20]. However, velocities as high as 4.4 cm s^−1^ were observed for the retrograde displacements of the particles. These values were higher than those observed using the magnetic pill in vivo and are most probably due to the shorter particle relaxation times in this study. However, it has to point out that the in vivo velocities were obtained at baseline conditions.

Moreover, higher retrograde velocities were observed in the DCM compared to the magnetic pill (Figure 10). Though, if the comparison was performed only for those in vitro conditions where the relaxation times of the particles used in this study and the magnetic capsule were comparable (i.e., 0.75% NaCMC, *w/w*), then the range of the in vitro velocities would be within the range of the in vivo observations (inset graph in Figure 10). However, there is no information about the fluid volume and the viscosity in vivo to confirm this. Therefore, it has to make clear that this comparison is to show at least that the data generated by the DCM is not too far from the physiological range, knowing, of course, the limitations of this exercise.

MRI measurements of the stomach and small bowel motility have been the subject of various studies since the late 1990s [37], developing to now include automated analysis of the data generated [38]. Some of these methods have shown the potential for clinical translation, e.g., motility alternations and patient-reported symptoms [39]. MRI of the motility of the colon has only recently started to be addressed [40]. The insights gained in colonic motility are novel and can link the in vivo physiological and un-disturbed environment to in vitro and in silico modeling of colon function, with particular application to our DCM model. Therefore, MRI is likely to generate more knowledge of the physiological ranges of fluid velocity in vivo in the human colon. Direct measurement of fluid flow inside the colon is, however, in its infancy. Previous work looked at backward and forward flow events in the stomach [41]. One study measured a mean flow velocity of 7.74 cm min^−1^ in the small intestine [42]. Direct flow velocity measurements in the colon are not available. A recent study used MRI tagging methods to monitor flow patterns in the healthy human colon prepared with a bowel cleansing preparation [36]. The authors observed flow events with velocities ≥288 cm min^−1^, as derived from the smearing in the deformation of the tagging lines. It has to be noted that this is related to the central axis flow and not within the haustra. Such high velocities were observed in DCM only at the retrograde direction (264 cm min^−1^ or 4.4 cm s^−1^, Figure 9) for the floating particle at 0.25% NaCMC (*w/w*) solution, confirming this fast-moving central retrograde “jet” observed in vivo [36]. The similar velocities to the in vivo observations are because the floating particle at low viscosity media, something that reflects the low viscosity of the enema normally used in in vivo studies, is located at the central axis (Figure 8), which is the area at which a particle will face the maximum fluid velocities. In addition, these fast retrograde flow velocities were also observed in vivo during the relaxation of the ascending colon wall (Video S2) after an antegrade wave ended at the hepatic flexure. DCM reproduced this phenomenon after a forward wave ended at the rigid siphon.

These initial experiences on gastrointestinal tract flow measurements with MRI are promising and, if developed and confirmed, could further inform the DCM to explore what would be the effect of different in vivo conditions to the performance of dosage forms within the ascending colon. However, mechanistic investigation of the ascending colon motility under fixed conditions in vivo is not easy as it requires access to this region to introduce fluid of known properties (i.e., density, viscosity) and volume. However, the colonic environment will dynamically change, and real-time monitoring of the changes in the luminal environment is not easy. Up till now, only one study provided real-time viscosity measurements in the stomach of locust bean gum-based meals using echo-planar MRI [43]. Similar work is required in order to increase our understanding of the colonic region. In particular, in the presence of dietary fibers, and how this will affect the performance of dosage forms due to the change in viscosity. This viscosity effect has shown to be important even for highly soluble drugs due to a decrease in the dissolution rate [44]. However, performing these experiments in vivo is time-consuming and costly, highlighting the possibility for the DCM to enable the acceleration of the screening of different drugs and formulations under multiple conditions. However, it should be clear that any further improvement of the DCM should be derived from in vivo knowledge of the colon gained by performing targeted-selective in vivo studies, and this work gives an example of how this can be done.

## 4. Conclusions

The DCM, a novel biomechanical computer-controlled model of the human proximal colon, informed by cine-MRI, was developed and used to simulate colon motility. The advantage of using the proposed in vitro DCM is that parameters such as motility pattern, viscosity, the volume of fluids, and properties of the tracking particles can be predetermined and controlled during the experiment. This allows assessment of the interplay between these parameters, i.e., how they affect the mixing and propulsion of the fluids in the proximal colon. In addition, the fact that DCM motility can be modified to reflect different in vivo conditions (baseline, stimulated and/or disease) addresses the unmet need for biorelevant-mechanistic in vitro models allowing the development of methods capable of achieving an IVIVE or IVIVC [45].

Different results can be obtained in terms of velocities and residence times by changing the particle used as a reference for analyzing the fluid motion. In general, high velocities and longer retrograde displacements were observed for the floating particle, which is also controlled by the viscosity of the fluids. These differences also reflected how accurately the particle describes the fluid motion since short relaxation times were obtained for different viscous media. In addition, it seems that the viscosity promotes the propulsion of fluids, provided that the particle has the desired location with respect to the contraction point. However, the limitations of the proposed in vitro model, addressed in the results and discussion section, should carefully be considered when extrapolating the findings of this work to in vivo conditions.

This work could be used to examine the behavior of different dosage forms within the colon lumen, with particular reference to the impact of particle shape and buoyancy on drug release. The DCM can also have applications in the development of modified release formulations targeting the colon, where existing models do not replicate the anatomy and flow, thereby having limited dissolution capacity and unknown interactions between the drug’s physicochemical characteristics, fluid dynamics, and possible food effects.

## Figures and Tables

**Table 1:** Parameters of the human caecum-ascending colon wall motility, derived from analysis of cine-MRI acquired at baseline and stimulated conditions, and the corresponding values used in the Dynamic Colon Model (DCM) motility.

Condition	N ^1^	Motility Index (Segment x s)	No of Waves ^2^		Travel Distance (cm)		Travel Velocity (cm s^−1^)		Occlusion Degree (%)	Occlusion Velocity (cm s^−1^)
			A	R	A	R	A	R		
Baseline	240	120 ± 50 *	1 *	-	3.9 *	-	0.98 *	-	18 ± 10 *	0.14 ± 11 *
Stimulated	720	320 ± 138 *	9 ± 1 *	2	5.3 ± 1.4 *	6.6 ± 2.2	2.2 ± 3.3 *	2.2 ±1.8	59 ± 18 *	3.6 ± 0.17 *
DCM	-	240	4	-	20	-	2	-	40	1.6

^1^ Number of MRI images analyzed; ^2^ Number of waves detected within the acquisition period (2 min); A: antegrade waves; R: retrograde waves; * significant statistical difference of the colonic motility between baseline and stimulated conditions (*p* < 0.05).

**Table 2:** Rheological properties of the fluids used in PEPT experiments as well as relaxation times and range of the residence times of the floating and neutrally buoyant particle along the Dynamic Colon Model tube obtained in different viscous media.

Fluid	%NaCMC (*w/w*)	*μ*_A_ (m Pa s)	*K* (Pa s^-n^)	*n*	Re	*t*_o_ (s)			Residence Times (s)	
									Floating Particle	Neutrally Buoyant Particle
L *	0.25	8	0.04	0.9	80	0.059 ^+^	0.065 ^++^	0.378 ^+++^ 0.178 ^++++^	25 ^a^–125 ^b^	50 ^a^–300 ^b^
M *	0.50	106	0.20	0.7	5.6	0.004 ^+^	0.005 ^++^	0.028 ^+++^ 0.013 ^++++^	50 ^a^–125 ^b^	150 ^a^–300 ^b^
H *	0.75	200	0.83	0.6	0.9	0.002 ^+^	0.003 ^++^	0.015 ^+++^ 0.007 ^++++^	100 ^a^–125 ^b^	175 ^a^–300 ^b^

^a^ The low values correspond to the residence times observed in the 1st unit of DCM tube; ^b^ The high values correspond to the residence times obtained in the last unit of the tube; ^+^ *t*_o_: particle relaxation times (s) of the floating particle; ^++^ *t*_o_: particle relaxation times (s) of the neutrally buoyant particle; ^+++^ *t*_o_: particle relaxation times (s) of magnetic pill used in Hiroz et al. (2009); ^++++^ *t*_o_: particle relaxation times (s) of magnetic pill used in Mark et al [21] L (Low viscosity, 8 mPa s), M (Medium viscosity, 100 mPa s), and H (High viscosity, 200 mPa s).

## Supplementary Materials

The following are available online at https://www.mdpi.com/1999-4923/12/7/659/s1, Figure S1: Designing the DCM model, Figure S2: The DCM system, Figure S3: Time courses of membrane oscillations, Figure S4: Calibration of the hydraulic system, Figure S5: Interface of the software used to control and synchronize the stepper motors of the hydraulic system, Figure S6: DCM-PET configuration, Figure S7: k/Plots of y and x-axes of the floating and neutrally buoyant particle for all the individual passes of the tracer along the DCM tube, Figure S8–S10: Plot of individual passes of the (a) floating particle and (b) neutrally buoyant particle along the DCM tube (x-axis), Video S1: cine-MRI of human ascending colon under baseline conditions, Video S2: cine-MRI of human ascending colon under stimulated conditions (oral PEG electrolyte bowel preparation), Video S3: cine-MRI of human ascending colon under stimulated conditions (maltose bowel preparation).
