# Supplementary material for: Dynamic Colon Model (DCM): A Cine-MRI Informed Biorelevant In Vitro Model of the Human Proximal Large Intestine Characterized by Positron Imaging Techniques
Source: Pharmaceutics. 2020 Jul 13;12(7):659. doi: 10.3390/pharmaceutics12070659 (PMC7407282; doi:10.3390/pharmaceutics12070659)
Supplement: Supplementary file 1 [file pharmaceutics-12-00659-s001.zip › pharmaceutics-831441-supple.docx]

Supplementary Materials: Dynamic Colon Model (DCM): A Cine-MRI Informed Biorelevant In Vitro Model of the Human Proximal Large Intestine Characterized by Positron Imaging Techniques

Konstantinos Stamatopoulos *, Sharad Karandikar, Mark Goldstein, Connor O’Farrell,
Luca Marciani, Sarah Sulaiman, Caroline L. Hoad, Mark J. H. Simmons and
Hannah K. Batchelor

The Dynamic Colon Model (DCM)

Examination of the MRI images demonstrated that the spacing of the haustra along cecum-ascending region was not constant. However, to simplify the design of the model, the number and the width of the haustra were fixed based on average values. The width of the haustra was 20 mm whereas the thickness of the semilunar folds and taenia coli was 2 mm and 5 mm, respectively [24]. Figure 1 shows the translation of the MRI images to a 3-D model of a single unit consisting of three haustra in triangular configuration (Figure S1).


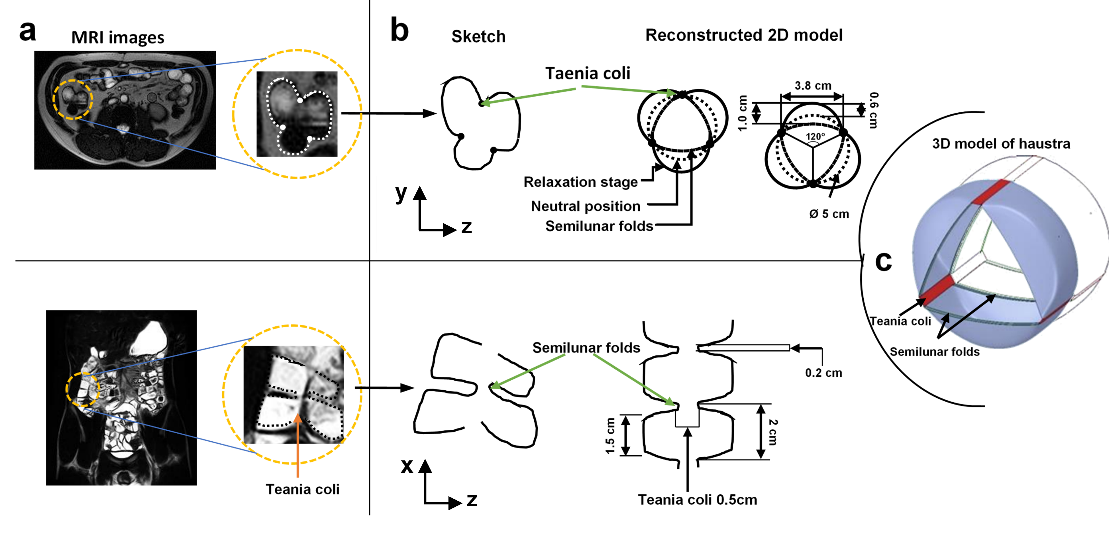


**Figure S1.** Designing the DCM model. (**a**) Transverse and coronal MRI images of the human caecum – ascending colon; (**b**) Haustra geometry model; (**c**) 3D model of the haustra.

The DCM is comprised of 10 haustral units (Figure 2A) with a total length of 200 mm (209 ± 47 mm length of cecum-ascending region in human [24]). The total volume of this tubular model is 290 cm3, which is within the physiological values with range 76–390 cm3 [25]. A 3-D printed rigid siphon was placed at the end of the biomechanical model representing the hepatic flexure. An acrylic based semi-spherical piece was machined to reproduce the curving shape of the cecum. A flat membrane was placed to separate the cecum from the main tube. This is also used to impart caecal motion. Each unit was connected to a syringe which was controlled by a stepper motor. Backward and forward motions of the syringe cause inflation and deflation of the membrane. Synchronization of the units was performed with using a computer – controlled hydraulic system (Figure 2B).

The hydraulic system was calibrated by correlating syringe displacements and membrane movements. A camera was used to capture images of the cross section of the DCM unit at 50 frames per second (fps) (Figure S1). The images were used to determine the degree of luminal occlusion (i.e. reduction of the cross-section area of the DCM unit) and the occlusion rate with respect to different travel distances and rates of travel of the syringe (Figure S2). The syringe movements were controlled by individual stepper motors being driven by Nanopro software (Nanotec Electronic GmbH & Co. KG, Germany).

After calibration of a single configuration (i.e. stepper motor – syringe – DCM unit), ten replicas were assembled into a framework (Figure 3C). The motors were connected in parallel using a controller (SMCI12, Nanotec Electronic GmbH & Co. KG, Germany) for each motor. The software used to control the hydraulic system was developed by the Biosciences workshop at the University of Birmingham (Figure S3). After assembling of the entire DCM tube, all the units were tested with the same occlusion and expansion speed, to assure that all units were calibrated correctly. Any inconsistency, e.g. higher occlusion degree for the same syringe displacement, was adjusted using the release valve located under each syringe (Figure 2B).


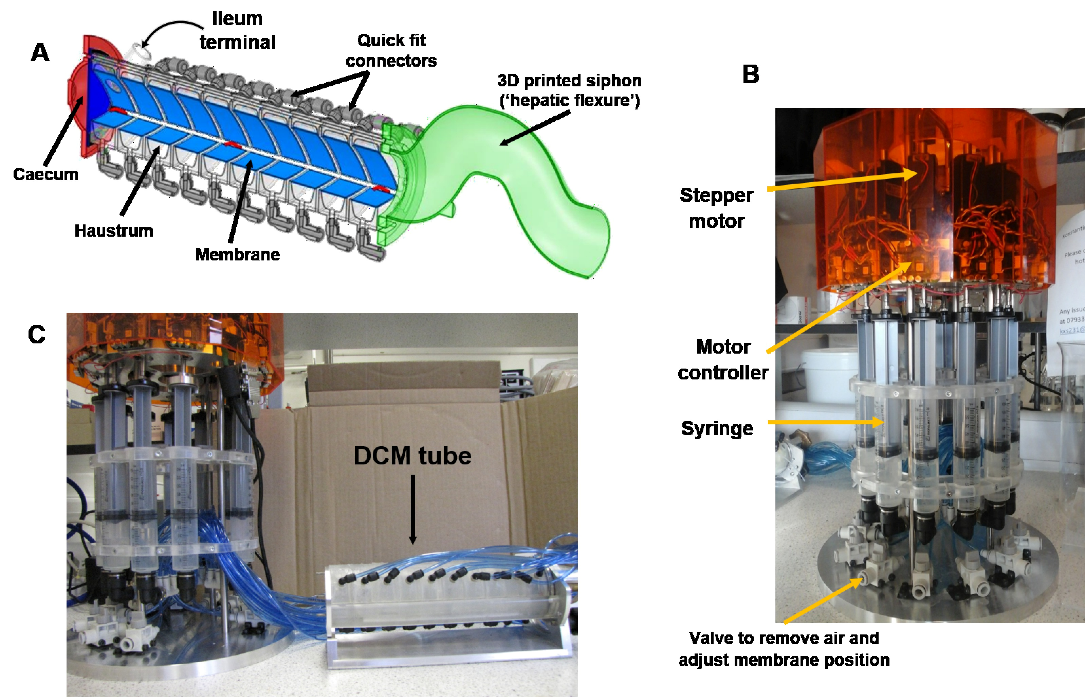


**a**

**b**

**c**

**Figure S2.** The DCM system. (**a**) 3D model of the biomechanical Dynamic Colon Model of human proximal colon with focus on caecum – ascending region; (**b**) The computer – controlled hydraulic system used to control the motion of the membrane of the Dynamic Colon Model (DCM); (**c**) DCM tube connected to hydraulic system.

**
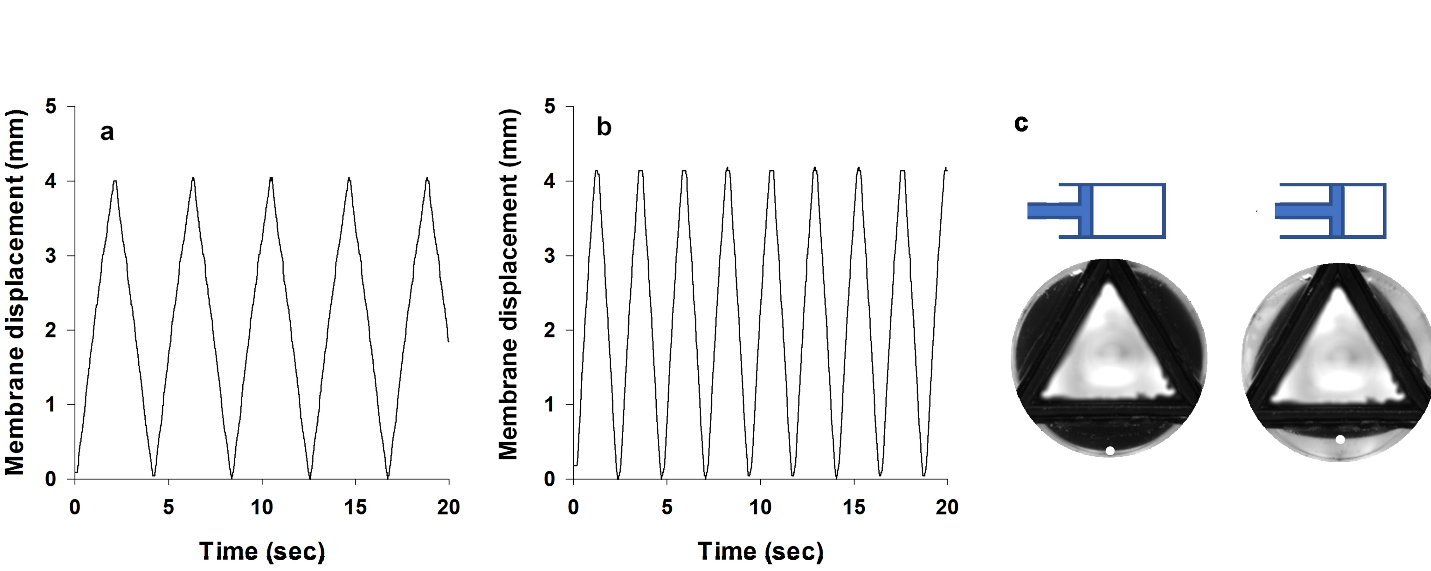
**

**Figure S3.** Time courses of membrane oscillations. (**a**) 1mm s-1; (**b**) 2 mm s-1 syringe travel speeds for fixed travel distance (10 mm); (**c**) The white dot on the cross section image of the DCM unit shows the reference point used to measure the travel distance of the membrane; a camera used at 50 fps to capture images of the cross section of the DCM unit during membrane’s oscillations.


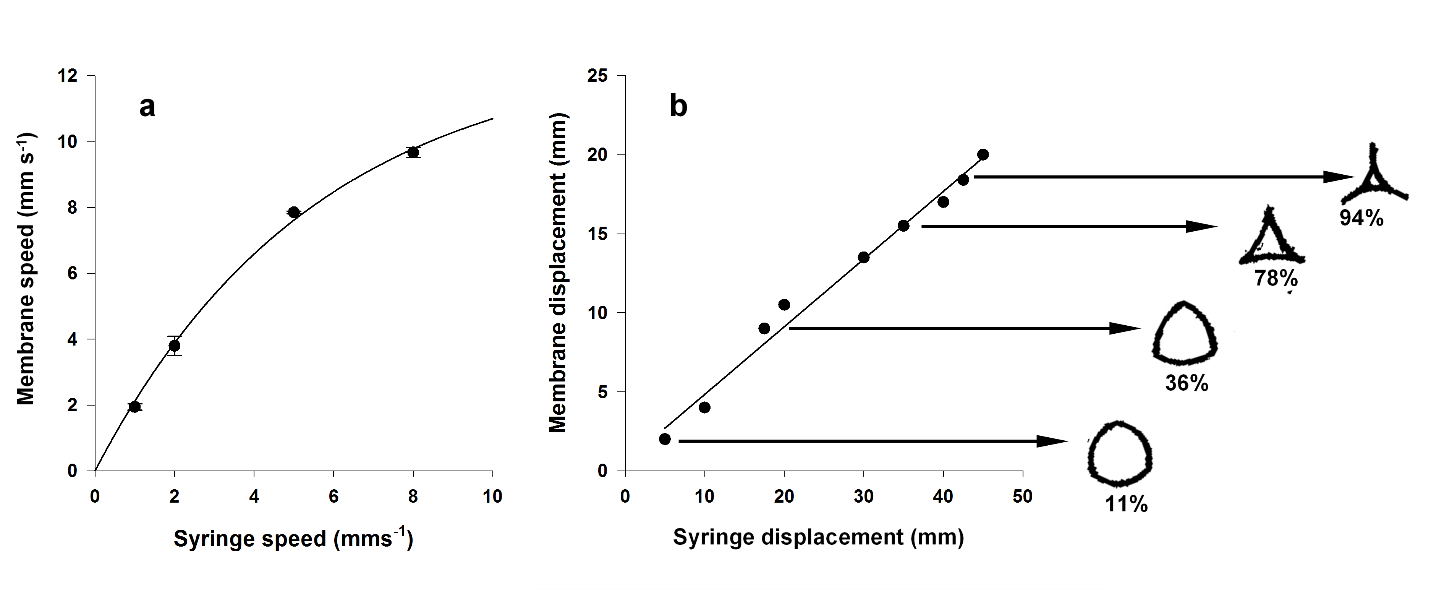


**Figure S4.** Calibration of the hydraulic system; (**a**) plot of syringe speeds (mm s^−1^) and the corresponding membrane speed displacement (mm s^−1^); b) plot of syringe displacements (mm) and the corresponding membrane displacement (mm), indicating also the % reduction of the cross-section area of the DCM unit. Standard deviation bars for the membrane travel speed (*n* = 3).


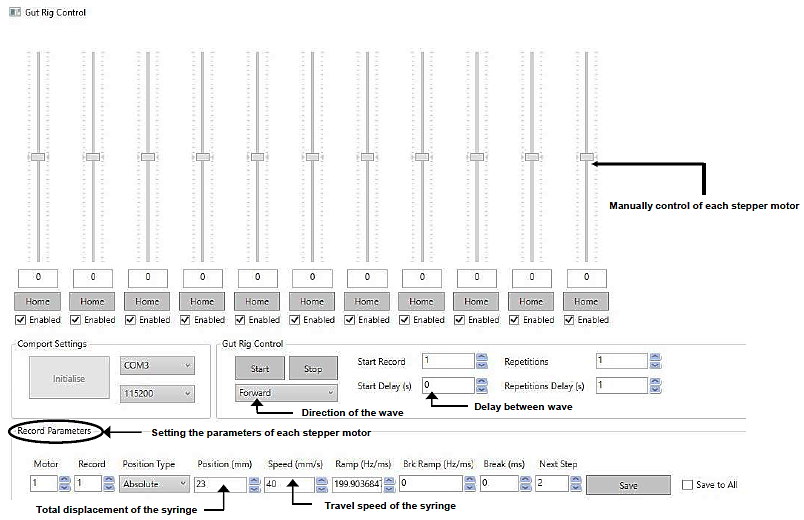


**Figure S5.** Interface of the software used to control and synchronize the stepper motors of the hydraulic system.


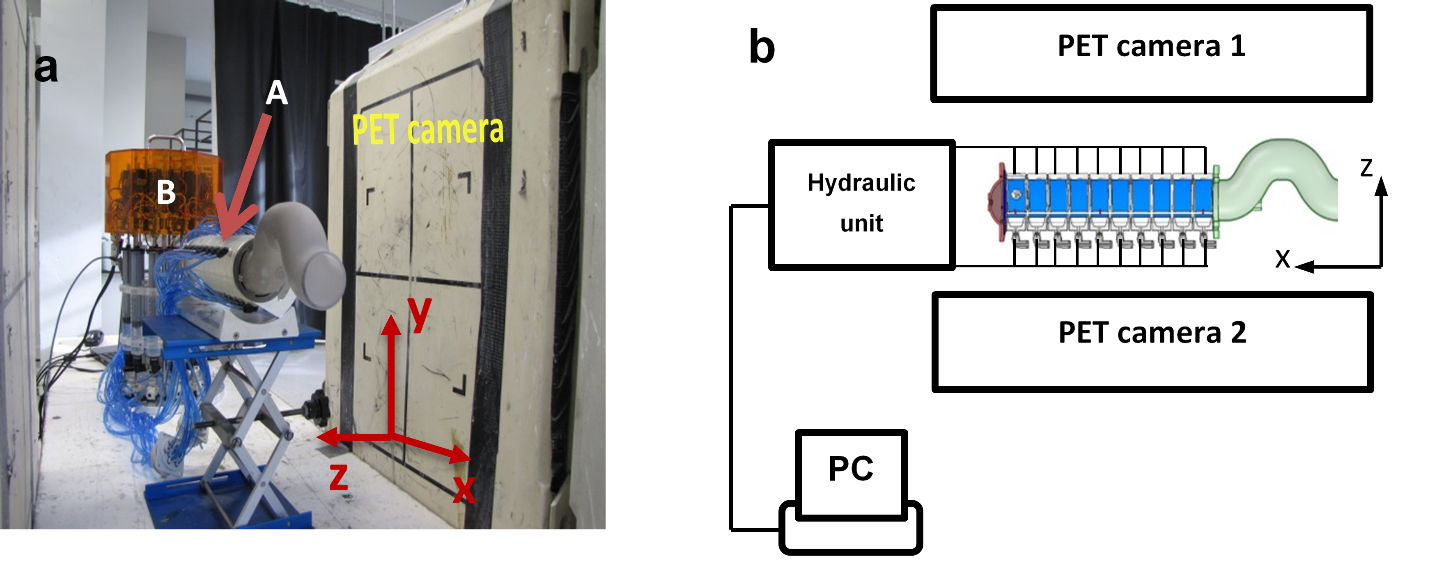


**Figure S6.** (**a**) DCM tube (A) in between PET cameras coupled with the hydraulic system (B); (**b**) schematic representation of the DCM – PET configuration.


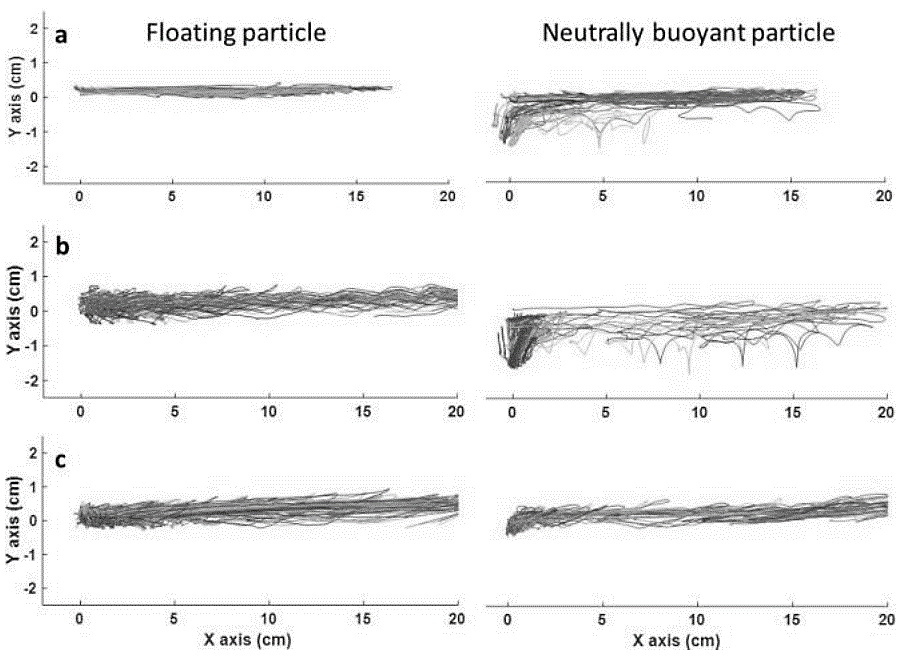


**Figure S7.** Plots of y and x axes of the floating and neutrally buoyant particle for all the individual passes of the tracer along the DCM tube. (**a**) 0.25%; (**b**) 0.5%; (**c**) 0.75% NaCMC (*w*/*w*).


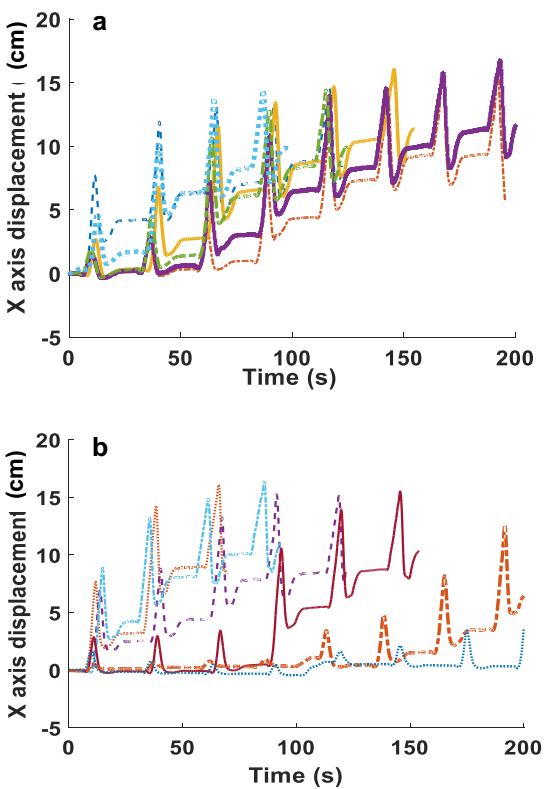


**Figure S8.** Plot of individual passes of the (**a**) floating particle and (**b**) neutrally buoyant particle along the DCM tube (x axis) in 0.25% NaCMC (*w*/*w*); every line represents one pass whereas each peak represents the motion of the particle in antegrade or retrograde direction after a single wave of haustral contractions. The plateau between the peaks is the time delay of 10 s before the next wave.


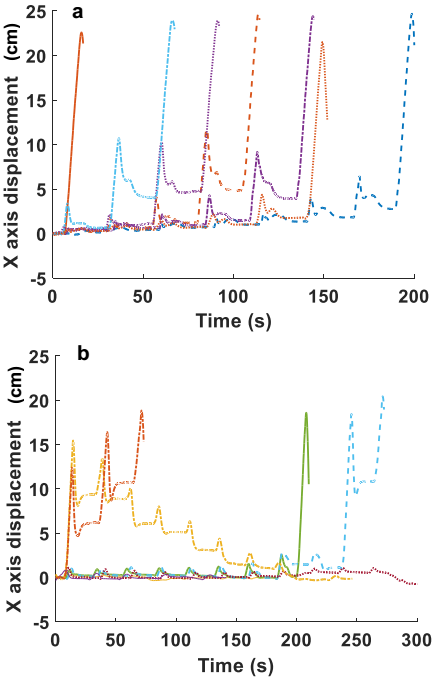


**Figure S9.** Plot of individual passes of the (**a**) floating particle and (**b**) neutrally buoyant particle along the DCM tube (*x* axis) in 0.50% NaCMC (*w*/*w*); every line represents one pass whereas each peak represents the motion of the particle in antegrade or retrograde direction after a single wave of haustral contractions. The plateau between the peaks is the time delay of 10 s before the next wave.


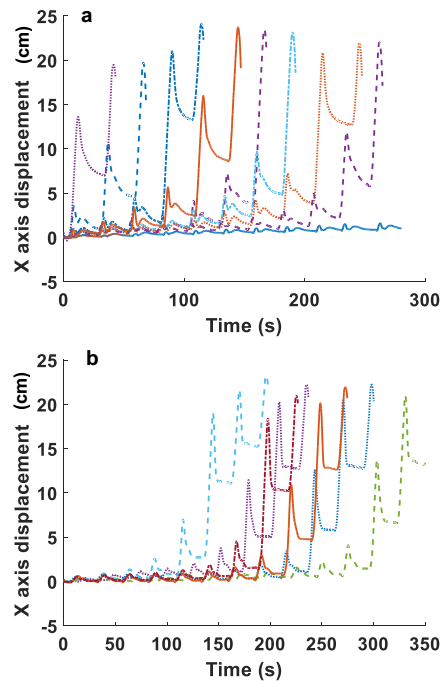


**Figure S10.** Plot of individual passes of the (**a**) floating particle and (**b**) neutrally buoyant particle along the DCM tube (*x* axis) in 0.75% NaCMC (*w*/*w*); every line represents one pass whereas each peak represents the motion of the particle in antegrade or retrograde direction after a single wave of haustral contractions. The plateau between the peaks is the time delay of 10 s before the next wave.
